# Uveal colobomas with pseudo-duplication of the optic disc in both eyes: imaging by ultra-widefield swept-source optical coherence tomography angiography: a case report

**DOI:** 10.1186/s13256-024-04676-z

**Published:** 2024-08-12

**Authors:** Sizhu Chen, Xinyue Liu, Jie Zhong

**Affiliations:** Department of Ophthalmology, Sichuan Provincial People’s Hospital, School of Medicine, University of Electronic Science and Technology of China, Chengdu, China

**Keywords:** Pseudo-duplication of the optic disc, Uveal colobomas, UWF-SS-OCTA, Case report

## Abstract

**Background:**

Duplication of the optic disc is a rare phenomenon. Differentiating between true duplication and pseudo-duplication requires a comprehensive set of diagnostic procedures. Advancements in imaging provide new insights into this condition.

**Case presentation:**

This report details a unique case involving an 8-year-old Han Chinese girl diagnosed with uveal colobomas and pseudo-duplication of the optic disc in both eyes. The extensive multimodal examination included assessments of vision, fundus examination, fundus photography, B-scan ultrasonography, ultra-widefield swept-source optical coherence tomography angiography, fundus fluorescein angiography, fundus autofluorescence, and magnetic resonance imaging.

**Conclusion:**

Ultra-widefield swept-source optical coherence tomography angiography proves to be a vital tool for examining and monitoring uveal colobomas with pseudo-duplication of the optic disc.

## Background

Double optic disc, a rare phenomenon, is categorized into true duplication and pseudo-duplication. In 1914, a case of optic nerve doubling was documented at autopsy, though earlier reports lacked comprehensive fundus examination details. A review of English literature on double optic disc cases reveals a clear differential diagnosis [1–8] for pseudo-duplication associated with uveal coloboma.

Colobomas in both eyes is uncommon [9]. Pseudo-duplication is typically linked to choroidal defects arising from incorrect optic fissure closure during fetal development, affecting the iris, ciliary body, retina, and choroid optic nerve between 5–7 weeks of gestation (7–20 mm development stage) [10]. The pseudo-optic disc is characterized by well-defined, optic disc-like lesions with vascular and surrounding chororetinal atrophy, generally positioned below the normal optic disc [1]. Other causes included acquired pseudo-double optic disc from pathological myopia [2]. Optical coherence tomography (OCT) has facilitated the examination of pathological features in both true duplication and pseudo-duplication. This study introduces the use of Ultra-widefield swept-source optical coherence tomography angiography (UWF-SS-OCTA) to delineate a rare case of uveal colobomas with pseudo-optic disc in both eyes.

## Case data

**Fig. 1:**
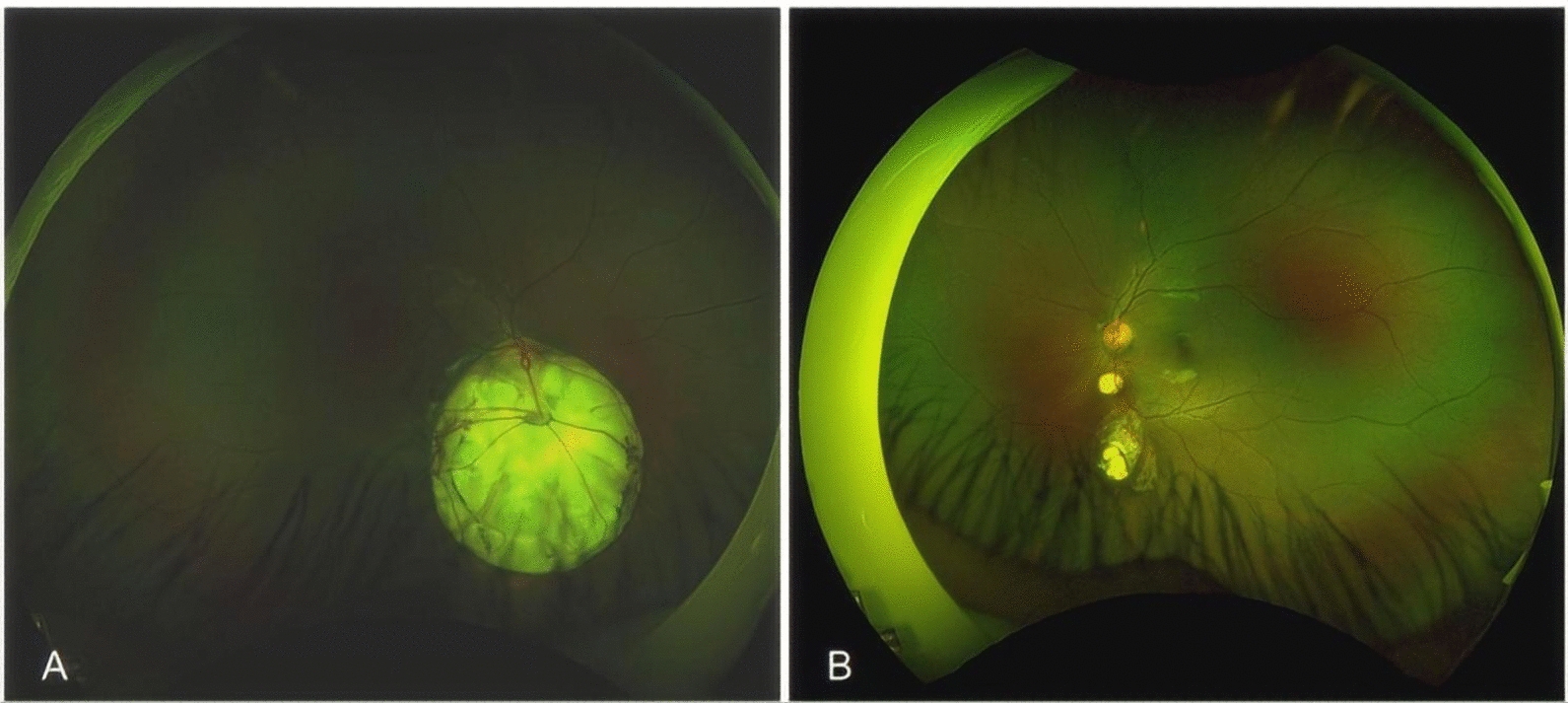
Fundus photography of the right eye (**A**) and the left eye (**B**)

**Fig. 2:**
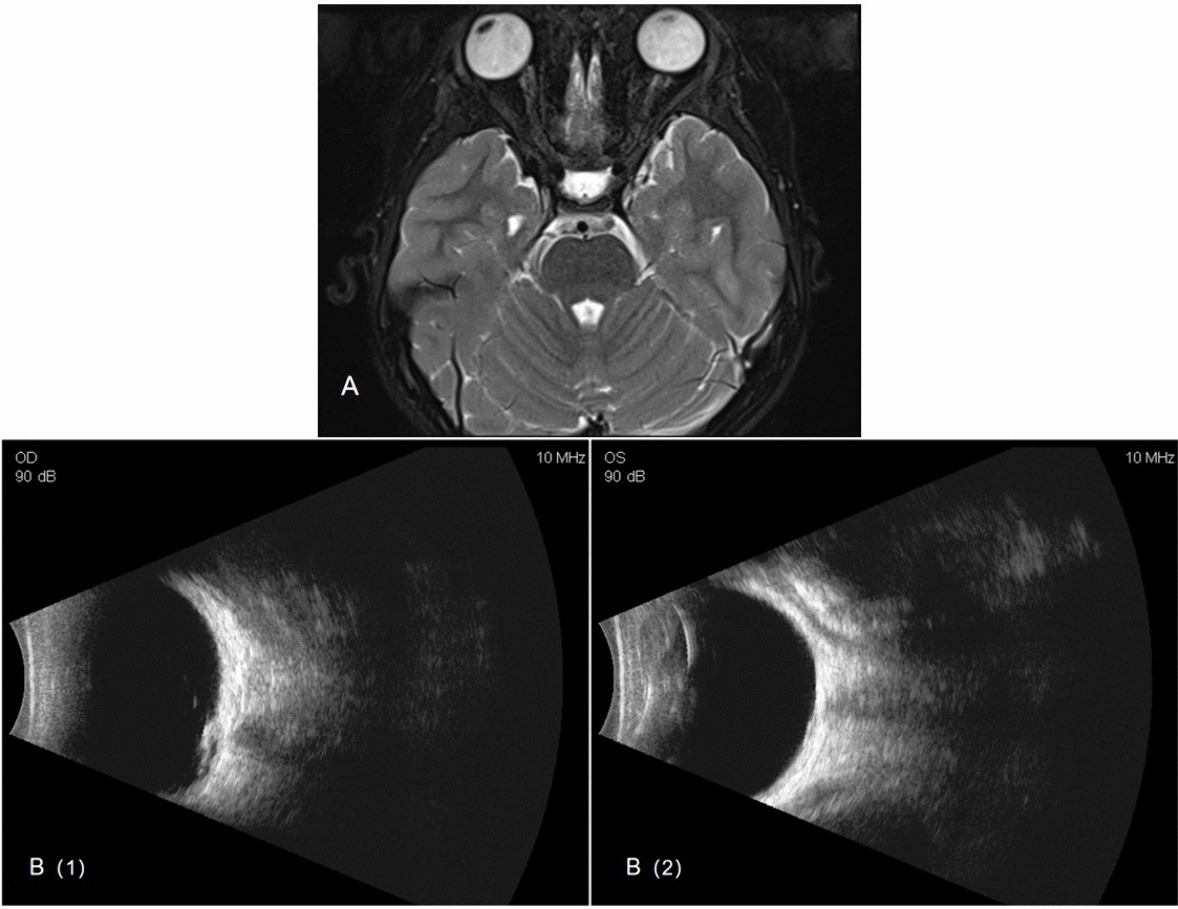
MRI (**A**) and B-ultrasound (**B**) of the eye showing a single optic nerve connected to both eyes

**Fig. 3:**
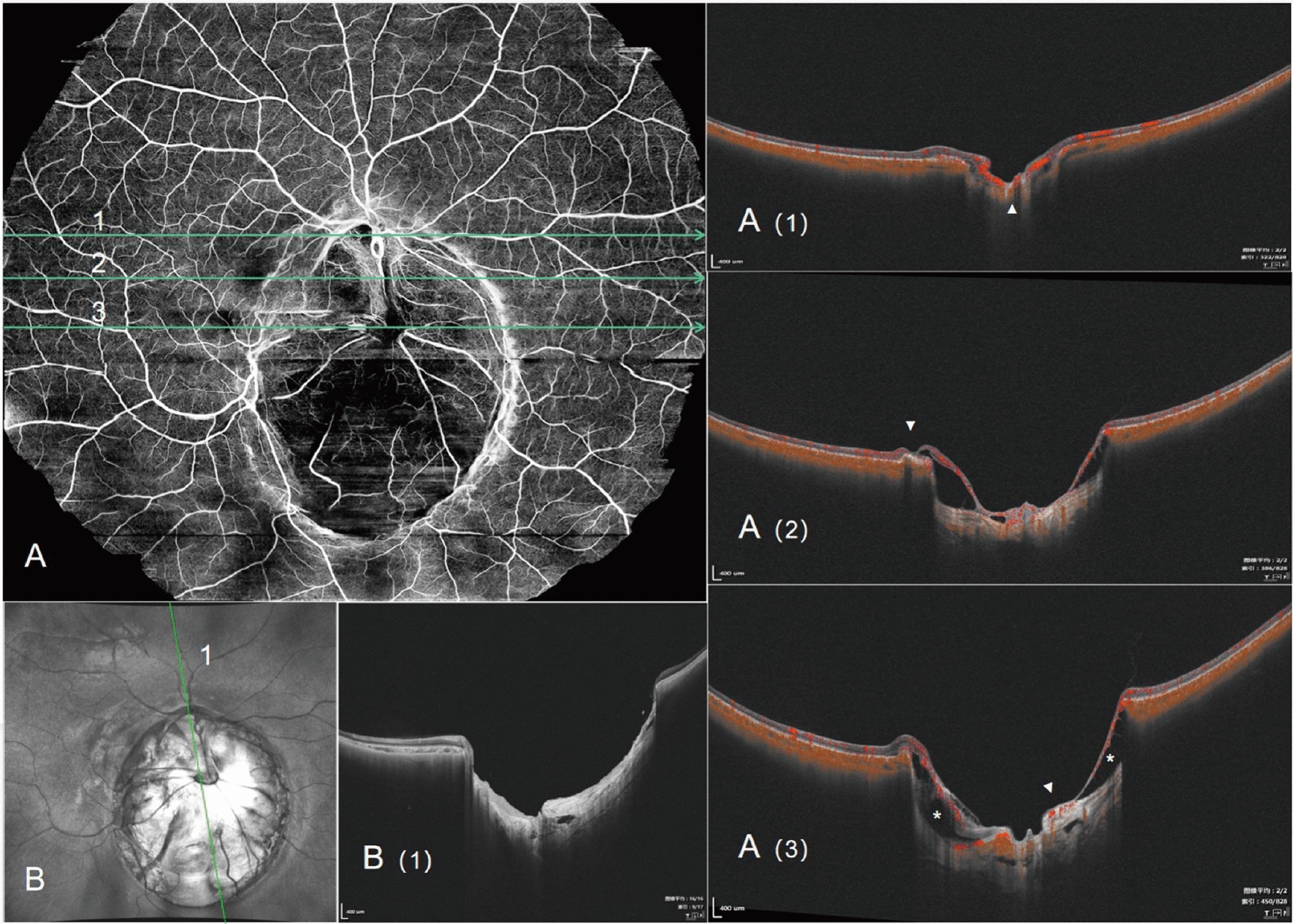
UWF-SS-OCTA image of the right eye fundus (29 mm × 24 mm). **A (1)** depicts the retinal nerve fiber layer as bundles descending through the upper optic disc, with blood flow signal (arrowhead) at the deep part of the optic disc. **A (2)** shows the B-scan image through the macula, featuring a central fovea structure (arrowhead); however, the macular area is at the margin of the colobomas and is partially affected. **A (3)** presents the B-scan image through the lower optic disc, where the star symbol denotes the detachment of the nerve fiber layer from the underlying sclera, and the arrow points to the thinning of the nerve fiber layer in an outward shape; the structure below the optic disc appears disordered. **B** En-face scan of the choroidal colobomas; **B (1)** shows a B-scan image through both optic discs, displaying the architecture of the optic tract in the upper optic disc, and a defective cavity area under the depression structure of the lower optic disc

**Fig. 4:**
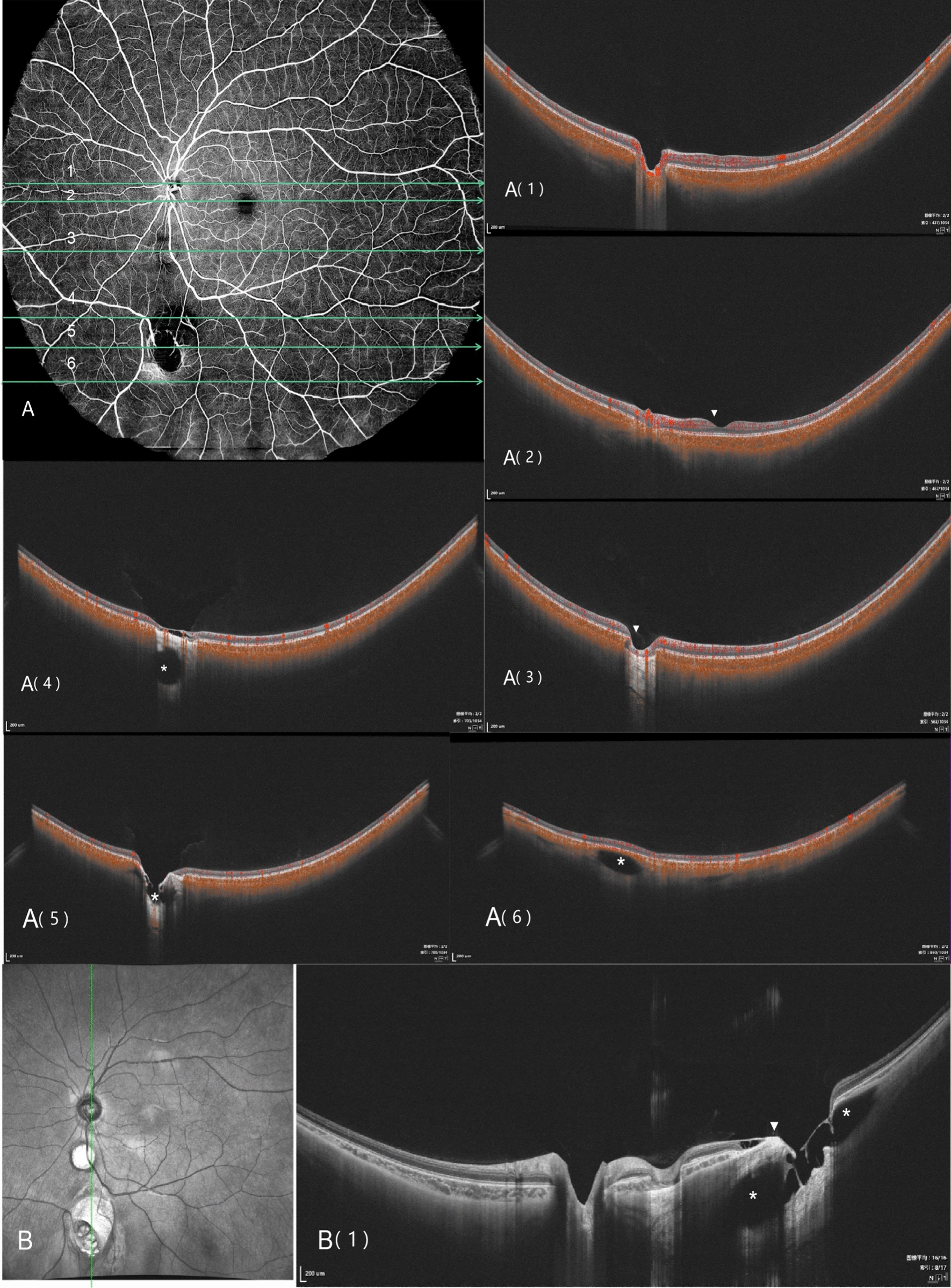
UWF-SS-OCTA image of the left eye, with B-scan images displayed in Fig. A (1–6); **A (1)** depicts the true optic disc, where the arrow highlights the optic nerve fiber bundles and deep blood flow signals; **A (2)** shows a B-scan image through the structurally intact macular area, with the arrow indicating the macular fovea; **A (3)** illustrates the choroidal defect area, where the choroidal layer is absent, yet the sclera remains intact, with the retina closely adhering to the sclera; **A (4)** presents a B-scan though the pseudo-optic disc, with the star marking a defect area encased by the sclera; **A (5)** features a B-scan highlighting the thin optic nerve fiber layer and detachment, without any optic tract-like structures, showing a star-marked defect area and the sclera indicated by an arrow; **A (6)** displays a B-scan below the pseudo-optic disc, with the star denoting a defect area within the layered sclera. **B** Enface structure diagram of the left eye, where **B (1)** captures a B-scan image of the two optic discs, with the arrow pointing to the optic nerve fibers and an ectatic coloboma beneath the pseudo-optic disc, resembling an inverted “Ω” shape

**Fig. 5:**
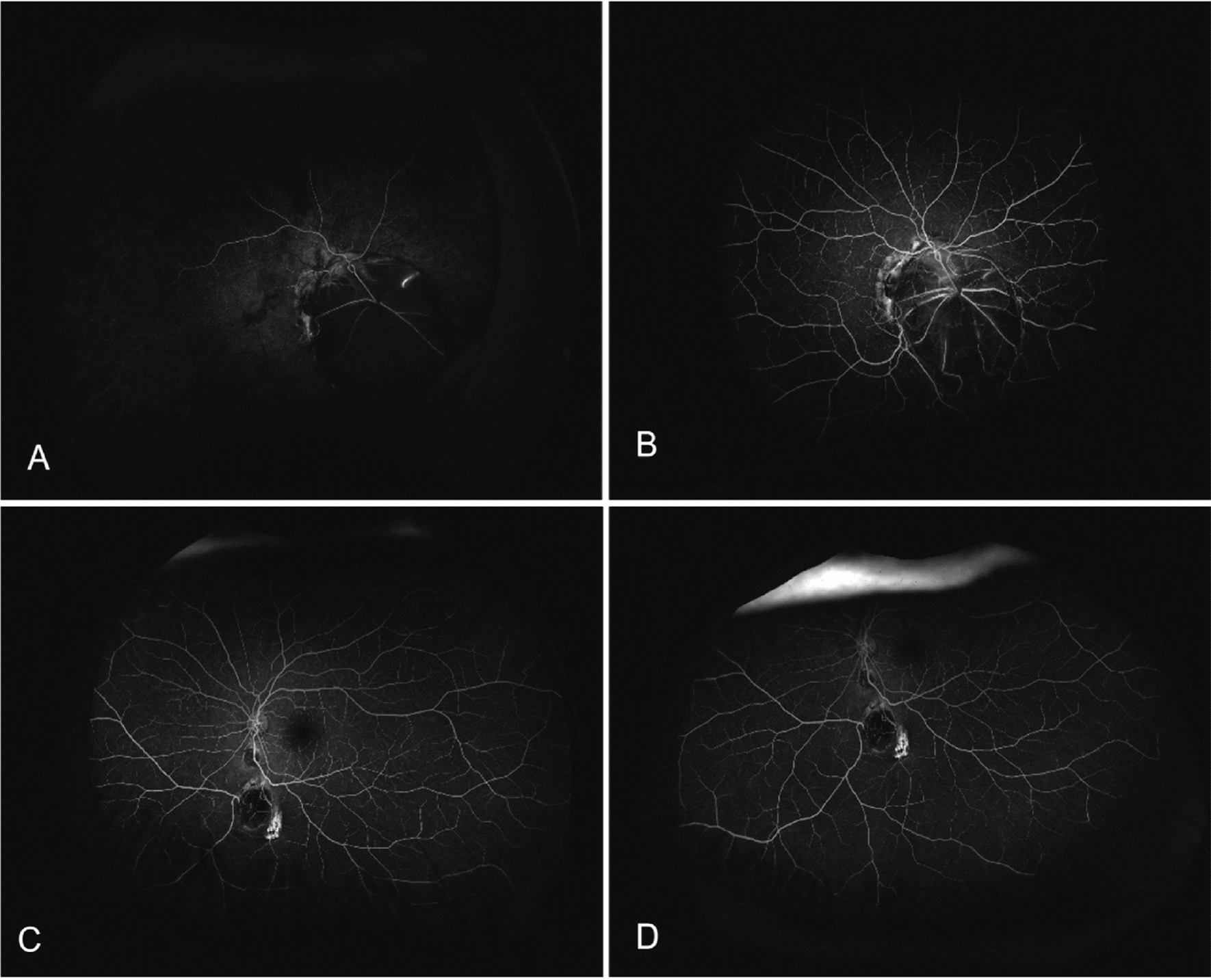
**A** shows the arterial phase of fundus fluorescein angiography in the right eye, with the 12-second image revealing the lower optic disc emitting inferiorly and superiorly, the upper vessel supply projecting the main superior temporal arteries, and a vessel bridging the two optic discs. **B** illustrates the right eye’s fluorescent contrast arteriovenous phase at 27 seconds, featuring upper and lower vascular systems. **C** and **D** depict the arteriovenous phase of fluorescein fundus imaging in the left eye at 8 seconds and 5 minutes 16 seconds, respectively, showing the upper optic disc emitting to the upper temporal and nasal branches, and the infranasal and infratemporal branches, with the vessels of the lateral nasal vein converging into the pseudo-optic disc

An 8-year-old Han Chinese girl was referred to our Sichuan Provincial People’s Hospital due to exotropia and diminished vision. Ophthalmologic evaluation revealed her best-corrected visual acuity to be 0.04 in her right eye (OD) with −1.50 defocus curve (DC) × 170° and 1.0 OS with −0.50DC × 175°. Intraocular pressure measured 11.2 mmHg in the right eye and 8.6 mmHg (1 mmHg = 0.133 kPa) in the left eye. She exhibited external strabismus in the right eye, but no ptosis or nystagmus was noted. The pupil diameter was 3 mm, it was reactive to light without iris defects, and both lenses and vitreous were normal. Fundus examination and photography (Fig. 1) indicated extensive choroidal colobomas affecting the macular in the right eye, with two distinct retinal vascular systems noted above the defect; a pseudo-double optic disc was observed in the left eye. The pseudo-optic disc, located approximately 6.5 mm beneath the true optic disc, was fed by a venous vessel from the nasal retina, accompanied by a distinct circular choroidal defect. B-ultrasound and orbital magnetic resonance imaging (MRI; Fig. 2) confirmed the presence of a single optic nerve in both eyes. UWF-SS-OCTA (VG200D, Intalight, Luoyang, China) and OCT analyses provided (Figs. 3, 4) detailed insights into the double optic disc, allowing measurements of the colobomas’ dimensions in the right eye and revealing a thinning nerve fiber layer around the pseudo-optic disc, even getting though the optic disc, which showed the nerve breaking off and deep blood flow imaging. The left eye displayed a normal macular, but in the right eye, there was a partially developed macular fovea at the edge of the coloboma. Fundus fluorescein angiography (FFA) indicated (Fig. 5) the right eye’s optic disc of choroidal colobomas emitting two main arteries upwards and two below, with an arterial vessel connecting the two discs. The left eye’s inferior nasal retinal vein was observed converging into the pseudo-optic disc. No systemic abnormalities such as deafness, mental retardation, microtia, or skeletal anomalies were identified. The family denied any genetic or familial history. The diagnosis was uveal colobomas with pseudo-duplication of the optic disc in both eyes. This study received approval from the Ethics Committee of Sichuan Provincial People’s Hospital and complied with the Declaration of Helsinki.

## Discussion

This report details a distinctive case of an 8-year-old Han Chinese girl diagnosed with uveal colobomas and pseudo-duplication of the optic disc in both eyes. Previous literature often utilized MRI, B-ultrasound, OCT, and FFA to differentiate between true and pseudo-duplication. Unlike prior studies, this is the first instance where UWF-SS-OCTA has been employed to examine the optic disc, retina, and sclera in children presenting with bilateral optic disc anomalies. This approach offers a benchmark for the rapid, non-invasive differentiation of true duplication and pseudo-duplication in an outpatient setting.

Optic disc duplication is categorized into true duplication and pseudo-duplication, with accurate differentiation between them being essential. In 1977, Jan Kaare Brink and colleagues [11] established criteria for true optic disc duplication: the additional optic disc must supply neural fibers, confirmed by interference filter photography and visual field tests. It should possess an independent or shared arterial vascular supply, verifiable through fundus fluorescein angiography (FFA). Computerized tomography should show two optic nerves emanating from the eyeball, and X-ray might reveal dual optic canals. In this case, although the child underwent most relevant tests, the absence of a visual field assessment and imaging evidence of a single optic nerve behind both eyes led to ruling out true optic disc duplication. Nonetheless, UWF-SS-OCTA observations indicated a distinct vascular supply to the pseudo-optic disc in both eyes, aligning with FFA results. Also noted were a thinning nerve fiber layer and a downward disconnection at the pseudo-optic disc, not forming a reflective bundle structure, and a continuous outer layer bulge at the inner scleral layer of the pseudo-optic disc, without creating an optic nerve canal. UWF-SS-OCTA proves effective for initial differentiation between true and pseudo-optic disc, requiring further confirmation in additional cases. Moreover, UWF-SS-OCTA detected an ectatic coloboma beneath the pseudo-optic disc, resembling an inverted “Ω,” especially apparent in Fig. 4B (1). Evaluating macular development is vital for visual acuity training in children, as illustrated in this case where the right eye, despite poor visual function, had a partially normal macular foveal structure.

Comparing the morphology of choroidal defects in both eyes of this case, along with their varied impacts on vision, it is evident that the eyes’ developmental processes were disparate. In the left eye, there was a distinct choroidal defect and scleral stratification between the superior and inferior optic discs, sparing the true disc, whereas the right eye displayed a substantial choroidal defect that included the optic disc. During the embryonic development of the optic nerve cup, the evagination of neuroectoderm forms the optic fissure, facilitating the entry of the hyaloid artery (primitive retinal artery) [12, 13]. The vascular branches from the pseudo-optic disc in the lower region of the left eye might have been positioned outside the optic nerve sheath at this time, while the binocular disc appearance in the right eye might result from a large posterior choroidal defect causing the non-merging of superior and inferior vessels. Prior research indicates that cells in the embryonic fissure are immature and susceptible to teratogenic damage [14]. Lingam Gopal and colleagues, in their classification of choroidal defects, suggested that each cell or cell cluster may have a vulnerability window, accounting for the inconsistent choroidal involvement observed [15]. Mistakes in the crucial timing and sequence of normal development lead to “continuous” or “skip lesions,” manifesting as unilateral or bilateral, symmetric, or asymmetric presentations [10].

Furthermore, while advancements have been made in understanding the embryological origins of follicular defects, the molecular and genetic mechanisms governing the closure of the optic fissure are not yet fully comprehended. Previous research has linked mutations in genes such as *CHX10*, *MAF*, *PAX6*, *PAX2*, *RX* (*RAX*), *SHH*, *SIX3*, *OTX2*, and *SOX2* to phenotypes associated with microphthalmia, anophthalmia, and coloboma (MAC). However, recent pediatric studies indicate that the vast majority (97%) of these defects are not attributable to known genetic mutations [16]. In this case, despite the absence of related systemic abnormalities such as deafness, intellectual delay, microtia, or skeletal anomalies, the child’s guardians opted out of genetic testing.

## Conclusion

UWF-SS-OCTA is an invaluable diagnostic and monitoring tool for congenital optic disc duplication and choroidal defects.

## Data Availability

Not applicable.

## Abbreviations

UWF-SS-OCTA: Ultra-widefield swept-source optical coherence tomography angiography
FAF: Fundus autofluorescence photography
FFA: Fundus fluorescein angiography
MRI: Magnetic resonance imaging
OCT: Optical coherence tomography
MAC: Microphthalmia, anophthalmia, and coloboma spectrum
